# Epidemiological and virological characteristics of seasonal influenza in the Western Pacific Region of the World Health Organization, 2011–2015

**DOI:** 10.5365/wpsar.2017.8.1.004

**Published:** 2017-03-28

**Authors:** Members of the WHO Western Pacific Region Global Influenza Surveillance and Response System

**Affiliations:** a Members of the WHO Western Pacific Region Global Influenza Surveillance and Response System are provided in the Acknowledgements.

## Introduction

Seasonal influenza is an acute viral infection that causes annual epidemics. The World Health Organization (WHO) estimates that the global disease burden of seasonal influenza is approximately one billion cases annually resulting in up to 500 000 deaths. (*1*) Epidemics are well defined as seasonal in northern and southern temperate climates with annual epidemics occurring in late winter or early spring. (*2*) In contrast, seasonal patterns in tropical and subtropical regions are less clear and tend to show more consistent levels of transmission year-round. (*3*, *4*)

The Western Pacific Region (WPR) of WHO comprises 37 diverse countries and areas with temperate and tropical climates inhabited by approximately 1.8 billion people in 2016. (*5*) Therefore, influenza is consistently circulating in variable locations in the Region. Collection and analysis of influenza surveillance data in WPR is particularly important due to evidence that novel influenza may emerge from persistent influenza reservoirs in the tropics and then spread to temperate regions. (*4*) A more comprehensive understanding of virological characteristics of influenza in this Region will contribute to improved predictions of emerging global influenza trends. For example, there is evidence that between 2002 and 2007 influenza viruses originating in several tropical WPR nations seeded seasonal A(H3N2) epidemics in temperate zones. (*6*)

The Global Influenza Surveillance and Response System (GISRS) is a WHO network that monitors global impact of influenza and evaluates potential pandemic risk of emerging strains. (*7*) GISRS also provides recommendations regarding viral strains in seasonal influenza vaccines, laboratory diagnostics and antiviral susceptibility. GISRS comprises 143 National Influenza Centres (NICs), six WHO collaborating centres (CCs), four Essential Regulatory Laboratories and other ad hoc laboratories. The WHO WPR has 21 NICs, three WHO CCs and two Essential Regulatory Laboratories. The NICs process thousands of specimens yearly of which a subset is sent to WHO CCs. (*8*) FluNet is a global platform that allows NICs and other GISRS-affiliated laboratories to upload virological information regarding number of specimens tested and resulting type, subtype and lineage. (*9*) It has been used in WPR since 1996. FluID, currently in a pilot phase, is a platform for sharing country epidemiological data that includes influenza-like illness (ILI) consultations by age group, total number of outpatients and total number of surveillance sites. (*10*)

Embedding influenza surveillance strategies within the Asia Pacific Strategy for Emerging Diseases (APSED) framework has supported significant advances in WPR influenza capacity. (*11*) Advances include improved surveillance systems, increased laboratory capacity and greater rates of reporting to FluNet. (*12*) An evaluation of the Region between 2006 and 2010 indicated increased sample submission and reporting through regional systems, particularly in response to the 2009 A(H1N1)pandemic. (*12*) In light of continued efforts to enhance influenza surveillance in the Region, this review provides an updated description of regional influenza surveillance systems focused on the epidemiological and virological characteristics of seasonal influenza. This review updates the results from the previous 2012 review, (*12*) considers how recommendations regarding surveillance strategy improvements have been implemented in the Region and discusses suggested future steps.

## Methods

### Data collection

Influenza surveillance data for 2011 to 2015 were collected from the 15 countries and areas with NICs in the WPR: Australia, Cambodia, China (including Hong Kong Special Administrative Region SAR), Fiji, Japan, the Lao People's Democratic Republic, Malaysia, Mongolia, New Caledonia (France), New Zealand, Papua New Guinea, the Philippines, the Republic of Korea, Singapore and Viet Nam.

Virological surveillance data included number of specimens collected, tested and influenza positive subtypes and lineages. These data were extracted from FluNet and confirmed by NIC focal points.

Descriptive and epidemiological data were collected from NICs via questionnaires developed in Microsoft Excel^®^. Questionnaires of descriptive surveillance system data and epidemiological data were collected from December 2015 through August 2016. The data collected included descriptive surveillance system information such as ILI case definitions and the numbers and descriptions of active surveillance sites as of 31 December 2015. Epidemiological data, including number of ILI cases by age group and geographic location of surveillance sites, were collected.

### Data analysis

Country-specific information on ILI surveillance systems, site numbers and case definitions were extracted from submitted questionnaires and compiled.

Virological and epidemiological data reported by epidemiologic week were combined into data per month. Data were graphed and grouped into four regions according to location and similarities in influenza patterns and to allow comparison with previously reported trends. (*12*) The groups were: (A) Northern temperate (Mongolia and the Republic of Korea); (B) China (including Hong Kong Special Administrative Region SAR); (C) Tropical (Cambodia, the Lao People's Democratic Republic, Malaysia, the Philippines, Singapore and Viet Nam); and (D) Southern (Australia, Fiji, New Caledonia (France), New Zealand and Papua New Guinea). When data were available, per cent ILI consultations were determined by taking monthly ILI consultations divided by total monthly consultations. Proportions for each group were calculated by adding ILI consultations or positive cases and dividing by total consultations or total specimens tested, respectively. Per cent positive data and total positive samples were also analysed by subtype and lineage, that is, A(H1), A(H3), A(other) and influenza B by year. Positive specimens from Japan were included in regional number of influenza positive cases.

## Results

### Surveillance systems

All 15 countries and areas reported data to FluNet during the reporting period. All countries and areas had ILI surveillance systems with variations in ILI case definition, type of surveillance systems and number of reporting sites (**Table 1**). At the time of reporting, Mongolia used the 2014 WHO case definition of acute respiratory infection with measured fever of ≥ 38 °C and cough with onset within the last 10 days. (*13*) Hong Kong Special Administrative Region SAR, Malaysia, Papua New Guinea, the Philippines and Viet Nam used the previous WHO ILI case definition of sudden onset of fever of > 38 °C and cough or sore throat in the absence of other diagnosis. (*13*) The others reported case definitions that required additional respiratory symptoms or a modified time frame of symptom onset. Minor case definition differences were reported among various ILI surveillance sites within Australia, Cambodia, Hong Kong Special Administrative Region SAR and New Zealand.

**Table 1:** Outpatient surveillance systems and case definitions, 2011–2015

Country	Surveillance system	ILI case definition
Australia	242 GPs and 69 EDs	Fever (≥ 38 °C), cough and fatigue (some within four days of presentation)
	Community online data collection and national call centre network	Cough and fever
Cambodia	7 hospitals	Sudden onset of fever ≥ 38 °C axillary within 5 days of presentation and fever at time of presentation, cough and/or sore throat in absence of other diagnosis
	3 health facilities	Sudden onset of fever ≥ 38 °C axillary and fever at time of presentation, cough and/or sore throat in absence of other diagnosis
China	562 hospitals and 408 network laboratories	Sudden onset of fever of > 38 °C and cough or sore throat
China, Hong Kong Special Administrative Region SAR	17 EDs	Cases with clinical diagnosis related to influenza, upper respiratory tract infection, fever, cough, sore throat or pneumonia
	64 outpatient clinics, about 50 GPs, 30 TCM clinics	Prior WHO definition*
Fiji	5 sentinel sites	Sudden onset of fever of > 38 °C plus cough and/or sore throat
Japan	Approximately 5 000 sentinel health facilities (approximately 3 000 paediatric and 2000 internal medicine health-care facility sites)	1) All of the following: sudden onset, high fever, upper respiratory tract inflammation, general malaise or other systemic symptoms, OR: 2) confirmation based on rapid diagnostic kit (regardless of symptoms).
Lao People's Democratic Republic	8 hospitals	Acute respiratory infection with fever of ≥ 38 °C and cough, with onset within last 7 days
Malaysia	239 sentinel outpatient sites	Prior WHO definition*
Mongolia	115 sentinel sites	2014 WHO definition**
New Zealand	Approximately 200 GPs	An acute respiratory tract infection with abrupt onset of at least two of the following: fever, chills, headache and myalgia
	Call centre network	One of 18 symptoms
Papua New Guinea	2 hospitals	Prior WHO definition*
Philippines	18 sites	Prior WHO definition*
Republic of Korea	200 sentinel clinics	Sudden onset of fever of > 38 °C and cough or sore throat
Singapore	18 polyclinics, 99 GPs	An acute respiratory infection with measured fever of ≥ 38 °C and cough or sore throat; with onset within the last 10 days
Viet Nam		
Hanoi	15 sentinel hospitals	Prior WHO definition*
Ho Chi Minh City	5 sentinel hospitals	Prior WHO definition*

*Prior WHO definition: a person with sudden onset of fever of > 38 °C and cough or sore throat in the absence of other diagnosis

**2014 WHO definition: acute respiratory infection with measured fever of ≥ 38 °C and cough; with onset within the last 10 days

ED: emergency department; GP: general practitioner; TCM: traditional Chinese medicine

Note: no data provided for New Caledonia (France)

Virological and epidemiological characteristics

For ILI patients that met the country case definition, the method for selecting cases for specimen collection varied among countries. Most commonly a set number of cases per week were selected for testing. All countries and areas also used various laboratory testing methodologies for influenza and subtype confirmation, including rapid test, reverse transcription polymerase chain reaction (RT–PCR), serology and virus culture.

The number of reported specimens tested for influenza between 2011 and 2015 tripled (**Table 2**), with over two million specimens reported to FluNet from WPR. Of positive specimens reported to FluNet from WPR, over 70% of the specimens were from China followed by Japan (11%) and Australia (5%). During this time period, 13% (*n* = 293 501) of processed specimens from countries and areas that submitted data on number of specimens tested were positive for influenza virus, with a yearly variation from 9% to 17% positive.

**Table 2:** Specimens tested and specimens positive for influenza by type/subtype/lineage in Western Pacific Region countries, 2011–2015

-	2011*	2012**	2013**	2014**	2015***
Number of specimens tested	217 975	339 229	456 918	583 004	652 124
Number of influenza positive specimens	24 382 (11.2%)	58 430 (17.2%)	42 251 (9.2%)	86 884 (14.9%)	81 554 (12.5%)
Seasonal influenza-positive specimens by type/subtype/lineage					
Influenza A total	14 994	31 617	33 921	63 301	55 471
A(H1)	10 487	963	15 855	22 909	4598
A(H3)	3460	28 542	17 064	38 519	50 588
A(other)	1039	2101	975	1862	1710
Influenza B total	9387	26 813	8309	23 556	26 136
B(Victoria)	728	8911	451	704	1368
B(Yamagata)	468	3837	1867	8641	16 593
B(lineage not determined)	8191	14 065	5991	14 211	8476

Note: total number of influenza positive specimens includes seasonal and non-seasonal influenza subtypes while influenza positive specimens by type/subtype/lineage includes only seasonal influenza

* 2011: Data from Australia, Cambodia, China, Fiji, the Lao People's Democratic Republic, Mongolia, Malaysia, New Caledonia (France), New Zealand, the Philippines, the Republic of Korea, Singapore and Viet Nam

** 2012–2014: Data from 2011 countries plus Hong Kong Special Administrative Region SAR

*** 2015: Data from 2012–2014 countries plus Papua New Guinea

Epidemiologic data were provided by 12 countries and areas. Fiji, New Caledonia, New Zealand and Papua New Guinea provided total number of weekly ILI consultations. Hong Kong Special Administrative Region SAR provided weekly ILI consultation rates per 1000 consultations by type of surveillance system (for example, general practitioners or traditional Chinese medicine practitioners). Australia, Cambodia, China, the Lao People's Democratic Republic, Malaysia, Mongolia, Singapore and Viet Nam provided data on number of ILI cases and total consultations.

**Fig. 1:**
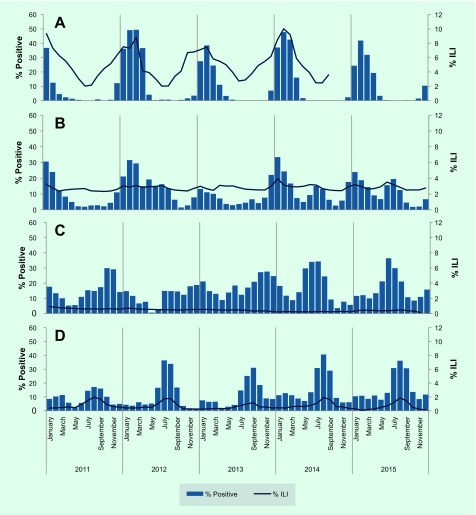
Proportion of specimens positive for influenza virus and proportion of consultations meeting influenza-like-illness (ILI) case definition by subregion within the Western Pacific Region, 2011–2015

Between 2011 and 2015, peaks in per cent ILI were generally consistent with per cent positive trends, particularly in the northern temperate and southern zones (**Fig. 1**). In Mongolia and the Republic of Korea, per cent ILI and per cent positive followed a northern temperate trend with yearly seasonal peaks occurring in the winter between January and March (Panel A, **Fig. 1**). Japan also exhibited the temperate northern hemisphere seasonality with distinct peaks in number of positive specimens seen at the beginning of each year (January or February). China (including Hong Kong Special Administrative Region SAR) demonstrated a bimodal influenza season with peak influenza activity between January and March consistent with the northern temperate season and secondary peaks occurring in June or July in some years (Panel B, **Fig. 1**). Seasonal trends were less evident for countries in the tropical region with occasional peaks several times a year. In 2014–2015, a peak around July appears to correspond with the secondary peak seen in China (including Hong Kong Special Administrative Region SAR) (Panels B and C, **Fig. 1**). The southern zone showed evidence of seasonal influenza transmission with highest levels of positive specimens and per cent ILI consultations reported between July and September each year (Panel D, **Fig. 1**).

Panel A: Northern temperate (% positive from Mongolia and the Republic of Korea and% ILI from Mongolia (2011–2014)); Panel B: China and Hong Kong Special Administrative Region SAR (% positive from China and Hong Kong Special Administrative Region SAR and% ILI from China); Panel C: Tropics (% positive from Cambodia, the Lao People's Democratic Republic, Malaysia, the Philippines, Singapore and Viet Nam and% ILI from Cambodia (2011–October 2015), the Lao People's Democratic Republic (2011–September 2015), Malaysia (2011–November 2015), Singapore (2011–2015) and Viet Nam (January–November 2015)); Panel D: Southern zone (% positive from Australia, Fiji, New Caledonia (France), New Zealand, Papua New Guinea and% ILI from Australia)

**Fig. 2:**
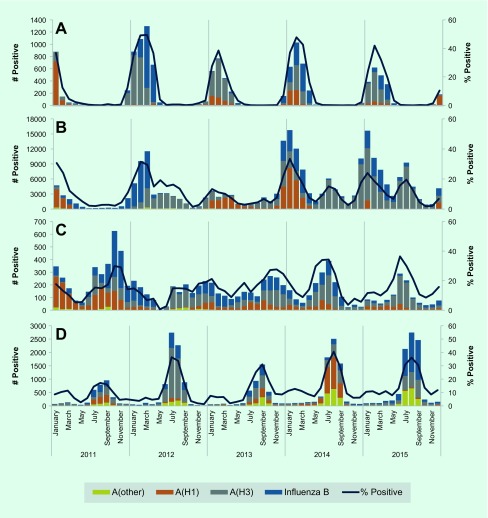
Number of influenza viruses by type/subtype and proportion of specimens positive for influenza virus in Western Pacific Region, 2011–2015

Influenza A was the predominant influenza type reported across all five years, for the entire WPR and by zone (**Table 2** and **Fig. 2**). In 2011, influenza virus A(H1) predominantly circulated during the first half of the year followed by B (lineage not determined) later in the year (**Table 2** and **Fig. 2**). In 2012, influenza B continued to circulate into the beginning of 2012 until influenza A(H3) began to predominate for the remainder of the year. From 2012 to 2015, the subtype A(H3) accounted for the largest proportion of the total influenza samples – ranging from 40% to 62%. From 2012 to 2015, A(H3) was the most frequently reported influenza subtype while secondary influenza subtypes and lineages varied during this time.

Panel A: Northern temperate (Mongolia and Republic of Korea); Panel B: China and Hong Kong Special Administrative Region SAR; Panel C: Tropics (Cambodia, Lao People's Democratic Republic, Malaysia, the Philippines, Singapore and Viet Nam); Panel D: Southern zone (Australia, Fiji, New Caledonia (France), New Zealand, and Papua New Guinea)

## Discussion

All countries and areas with NICs in WPR exhibited expected seasonal influenza prevalence and trends from 2011 to 2015. Advances in surveillance systems and laboratory capacity have been well documented over the past 10 years. There was a 10-fold increase in the number of ILI specimens tested between 2006 and 2015, driven predominately by increases in data submissions from China (including Hong Kong Special Administrative Region SAR). (*12*) This increase was likely due in part to increased awareness of the importance of specimen collection and submission following the A(H1N1) 2009 pandemic. (*12*) These data improve regional understanding of circulating viral subtype seasonal trends despite variations in laboratory and surveillance systems, case definitions and number of surveillance sites.

All 15 countries and areas surveyed have sentinel influenza surveillance systems in place. Since the last regional overview, ILI case definitions and number of surveillance sites have changed within many countries included in this review (see **Table 3**). The previous regional overview (2006–2010) reported that eight countries and areas used the WHO case definition. (*12*) In 2014, the official WHO case definition for ILI changed from sudden onset of fever of > 38 °C and cough or sore throat to a new case definition that removed sore throat from the definition and required symptom onset within 10 days of presentation. (*13*) In 2015, one country used the 2014 WHO case definition, five countries and areas reported the use of the previous WHO case definition, and the other countries reported use of alternatives (see **Table 1**). As changes in case definition have been shown to impact the sensitivity and positive predictive value of ILI sentinel surveillance, this should be taken into consideration when interpreting these results. (*13*)

**Table 3:** ILI case definitions and surveillance systems in the Western Pacific Region, 2006–2010 compared to 2011–2015

Year	Country	Surveillance system	ILI case definition
2006–2010	Australia	Approximately 25 GP clinics	Fever (≥ 38 °C), cough and fatigue
		69 EDs	Fever (≥ 38 °C) or feverish plus at least one of the following symptoms: cough or sore throat
		Community online data collection	Cough and fever
2011–2015		242 GPs and 69 EDs	Fever (≥ 38 °C), cough and fatigue (some within four days of presentation)
		Community online data collection and national call centre network	Cough and fever
2006–2010	Cambodia	8 hospitals	Sudden onset of fever of > 38 °C and cough or sore throat within 5 days
2011–2015		7 outpatient department hospitals	Sudden onset of fever ≥ 38 °C axillary within 5 days of presentation and fever at time of presentation, cough and/or sore throat in absence of other diagnosis
		3 health facilities	Sudden onset of fever ≥ 38 °C axillary and fever at time of presentation, cough and/or sore throat in absence of other diagnosis
2006–2010	China	2010: 556 sentinel hospitals and 411 network laboratories	Sudden onset of fever of > 38 °C and cough or sore throat
2011–2015		562 hospitals and 408 network laboratories	As above
2006–2010	Hong KongSAR	114 public and private outpatient clinics	Prior WHO definition*
2011–2015		17 EDs	Cases with clinical diagnosis related to influenza, upper respiratory tract infection, fever, cough, sore throat or pneumonia
		64 outpatient clinics, 50 GPs, 30 TCM clinics	Prior WHO definition*
2006–2010	Fiji	13 sentinel hospitals	Prior WHO definition*
2011–2015		5 sentinel sites	Sudden onset of fever of > 38 °C plus cough and/or sore throat
2006–2010	Japan	3000 paediatric and 2000 internal medicine sites	Sudden onset of fever of > 38 °C, upper respiratory infection and feeling tired
2011–2015		Approximately 5 000 sentinel health facilities (approximately 3 000 paediatric and 2000 internal medicine health care facility sites)	1) All of the following: sudden onset, high fever, upper respiratory tract inflammation, general malaise or other systemic symptoms, OR: 2) confirmation based on rapid diagnostic kit (regardless of symptoms).
2006–2010	Lao People's Democratic Republic	8 hospitals	Prior WHO definition*
2011–2015		8 hospitals	Acute respiratory infection with fever of ≥ 38 °C and cough, with onset within last 7 days
2006–2010	Malaysia	Approximately 600 government health clinics	Prior WHO definition*
2011–2015		239 sentinel outpatient sites	Prior WHO definition*
2006–2010	Mongolia	37 hospitals and 121 health centres	Prior WHO definition*
2011–2015		115 sentinel sites	New WHO definition**
2006–2010	New Caledonia (France)	2 hospitals and 7 health centres	Sudden onset of fever ≥ 38 °C (or shiver if temperature not available) and cough (or sore throat)
2006–2010	New Zealand	Approximately 101 sentinel GPs operating May–September	An acute respiratory tract infection with abrupt onset of at least two of the following: fever, chills, headache and myalgia
2011–2015		Approximately 200 GPs	As above
		Call centre network	One of 18 symptoms
2011–2015	Papua New Guinea	2 hospitals	Prior WHO definition*
2006–2010	Philippines	59 health centres and hospitals	Fever of > 38 °C and cough or sore throat. For children ≤ 3 years, fever of > 38 °C and cough, sore throat or runny nose
		18 sites	Prior WHO definition*
2006–2010	Republic of Korea	Approximately 800 sentinel sites	Sudden onset of fever of > 38 °C and cough or sore throat
2011–2015		200 sentinel clinics (since 2013)	Sudden onset of fever of > 38 °C and cough or sore throat
2006–2010	Singapore	18 government clinics, 98 GP clinics	Prior WHO definition*
2011–2015		18 polyclinics, 99 GPs	An acute respiratory infection with measured fever of ≥ 38 °C and cough or sore throat; with onset within the last 10 days
2006–2010	Viet Nam		
	Hanoi	15 sentinel hospitals	Prior WHO definition*
	Ho Chi Minh City	5 sentinel hospitals	Prior WHO definition*
2011–2015	Hanoi	15 sentinel hospitals	Prior WHO definition*
	Ho Chi Minh City	5 sentinel hospitals	Prior WHO definition*

* Prior WHO definition: a person with sudden onset of fever of > 38 °C and cough or sore throat in the absence of other diagnosis

**New WHO definition: acute respiratory infection with measured fever of > 38 °C and cough; with onset within the last 10 days

ED: emergency department; GP: general practitioner; TCM: traditional Chinese medicine

Note: no data provided for New Caledonia (France) 2011–2015

The proportion of outpatient visits for ILI followed expected trends in the northern temperate zone, China (including Hong Kong Special Administrative Region SAR) and the southern zone, with peak consultations occurring during the same months as peak per cent positive specimens (**Fig. 1**). Per cent ILI in the tropical zone was low and consistent throughout the year. Seasonal trends in circulating virus identified predictable temperate zone peaks and consistent tropical circulation similar to the previous regional overview. (*12*) However, in 2014 and 2015, both China (including Hong Kong Special Administrative Region SAR) and the tropics appear to exhibit more distinct seasonal patterns with a bimodal distribution in China (including Hong Kong Special Administrative Region SAR) and occasional sharp peaks in the tropics (Panels B and C, **Fig. 2**).

Improvements in tropical indicator-based surveillance for ILI over recent years indicate that more definitive determination of tropical seasonality may be possible in the near future. For example, in the American tropics a recent study has shown that 13 out of 16 countries in that region experience peak influenza transmission between April and September with smaller secondary epidemics. (*14*) The observed peaks were not as distinct as those found in temperate regions; however, initial patterns of predictable seasonality emerged. This evidence of influenza seasonality illustrates the importance of strong outpatient indicator-based surveillance systems and reporting for determining seasonality which may impact vaccine policy.

The 2012 report recommended advancement of the following three areas of influenza surveillance: (a) improving virological testing capacity, (b) improving communication through regional and global networks, and (c) defining regional burden of disease. (*12*) Advances were documented in all three areas. Virological testing capacity continues to be strengthened. The number of reported virological tests conducted on influenza specimens has steadily increased from 65 103 specimens in 2006 to 307 584 in 2010 (*12*) and 652 124 in 2015; some countries showed slight decreases in the amount of data submitted as they continue to optimize their surveillance systems. Although the increase in number of samples over time does not necessarily constitute system improvement, consistent specimen submission does indicate both improved capacity and continued viability of the system itself. Evidence from the WHO external quality assessment programme shows an increase in the number of laboratories in the Region participating in the programme and consistently good results from participating laboratories (personal communication). Continued efforts placed on quality laboratory testing will ensure an accurate understanding of influenza in the Region.

Communication in the Region and globally continues to improve with increased reporting by NICs to FluNet. Other platforms such as the biweekly influenza situation updates published by the WHO Western Pacific Regional Office and periodic journal articles illustrate how communication and collaboration within the Region is prioritized. Using data visualization technologies, an online regional influenza dashboard is under way to integrate laboratory and epidemiological data in near real-time and provide a more complete picture of regional influenza activity. Finally, significant progress in regional risk communication capacity in response to recent emerging events (for example influenza A(H7N9) in China, 2013 and Zika, 2016) also benefits influenza surveillance and response efforts. (*15*)

Influenza surveillance in the Region continues to advance, and efforts to determine burden of disease are ongoing. WHO guidelines recommend assessing burden from acute lower respiratory infection and/or severe acute respiratory infection surveillance. (*16*) Several WPR countries, including Cambodia, the Lao People's Democratic Republic, Mongolia and Viet Nam, have begun burden of disease estimates including sentinel site catchment population determination. These estimates will contribute to national, regional and global burden estimates and may support consideration of vaccination in high-risk populations.

### Conclusions and way forward

Successful collaborative efforts between 2011 and 2015 continue to outline influenza epidemiological and virological characteristics in WPR and improve data to support ongoing public health action. A geographically wide range of influenza circulation patterns, covered by an extensive outpatient surveillance network, indicated temperate and tropical trends similar to those reported previously. Moving forward, WPR countries and areas are encouraged to focus on continued virus sharing through global networks while strengthening event-based surveillance, risk assessment and decision-making capacities. In addition, prioritization of high-quality, representative surveillance data of both outpatient and hospitalized respiratory disease will allow, respectively, improved appreciation of seasonality and economic burden of disease estimates. Finally, such estimates will support national influenza vaccination policies in high-risk groups. Advances in these areas will allow the Region to remain vigilant in the face of the continued, unpredictable influenza threat and further support the critical use of influenza vaccines in vulnerable populations.
